# Combined Fried Frailty Scale and Mini Nutritional Assessment Identifies Cardiovascular Patients with Reduced Protein/Albumin Plasma Levels: A Cross-Sectional Study

**DOI:** 10.3390/nu17172786

**Published:** 2025-08-27

**Authors:** Julia Cieśla, Marcin Schulz, Michał Krawiec, Michał Janik, Paweł Wojciechowski, Iga Dajnowska, Dominika Szablewska, Jakub Bartoszek, Brygida Przywara-Chowaniec, Andrzej Tomasik

**Affiliations:** 1 Doctoral School, Medical University of Silesia, Poniatowskiego 15, 40-055 Katowice, Poland; 2 School of Medicine, Medical University of Silesia, Poniatowskiego 15, 40-055 Katowice, Poland; mail.schulz.od44@gmail.com (M.S.); s81066@365.sum.edu.pl (M.K.); d201296@365.sum.edu.pl (M.J.); s88354@365.sum.edu.pl (P.W.); s88499@365.sum.edu.pl (I.D.); s88468@365.sum.edu.pl (D.S.); s85584@365.sum.edu.pl (J.B.); 3 II Department of Cardiology, Faculty of Medical Sciences in Zabrze, Medical University of Silesia, Marii Skłodowskiej-Curie 10, 41-800 Zabrze, Poland; bprzywara-chowaniec@sum.edu.pl

**Keywords:** frailty, malnutrition, sarcopenia, elderly, biomarkers, Mini Nutritional Assessment, SARC-F

## Abstract

**Background:** Frailty syndrome significantly worsens the prognosis of elderly people. Sarcopenia and malnutrition are integral parts of aging, but there is a lack of knowledge about the overlap of these states, as well as their diagnostic methods and treatments. This study aimed to assess the malnutrition and sarcopenia scale in patients with frailty syndrome and the viability of evaluating the concentrations of the following potential biomarkers: albumin, total protein, and fibrinogen. **Methods:** A total of 170 patients >65 years were assessed for frailty using the Fried frailty scale. The risk of sarcopenia was measured using the SARC-F scale, and the risk of malnutrition was measured using the Mini Nutritional Assessment (MNA) scale. Serum albumin, protein, and fibrinogen levels were measured. The following fat-free body mass and respiratory parameters were measured: peak expiratory flow (PEF) and forced expiratory volume-one second (FEV1). **Results:** A total of 53 patients were classified as robust (31%), 96 as prefrail (57%), and 21 as frail (12%) according to Fried frailty criteria. Frail patients had significantly reduced serum albumin and increased fibrinogen compared to the prefrail and robust groups (*p* < 0.05). A total of 38% of frail patients were at risk of sarcopenia, and 52% were at risk of malnutrition. Frail patients had lower PEF and FEV1 values, with decreases in respiratory parameters correlating with fat-free body mass and muscle strength. **Conclusions:** Frail patients are at substantial risk of malnutrition and sarcopenia. The MNA and SARC-F scales, combined with routine screening of elderly patients with frailty, may effectively identify patients with the highest risk.

## 1. Introduction

Frailty syndrome is defined as a decrease in the body’s reserves and a simultaneous increase in susceptibility to endogenous and exogenous stress factors [1]. The importance of frailty is increasing with the aging global population. At the same time, risk factors for developing frailty include a wide range of sociodemographic, clinical, lifestyle-related, and biological factors [2]. Moreover, frailty is not simply aging, but a progressive process that worsens quality of life, functionality, and increases overall risk [3]. Patients defined as frail have a significantly increased risk of falls, bone fractures, disability, dementia, hospitalization, and death compared to their healthy peers [4].

An essential aspect of developing knowledge about frailty syndrome is the search for its biomarkers. Many candidates have been described so far, among metabolic, hematological, and immunological molecules [5], including tumor necrosis factor alpha, interleukin 6 [5], C-reactive protein, hemoglobin, free testosterone, and 25-hydroxyvitamin D [6]. Due to the multifaceted nature of frailty, identifying reliable biomarkers poses a significant challenge. However, to better understand the pathophysiology of frailty and explore targets for potential treatment, finding biomolecules associated with frailty seems to be one of the most critical goals in modern geriatrics.

The integral elements coexisting with frailty syndrome are sarcopenia and malnutrition [7], which, together with cachexia, are defined as symptoms of tissue loss. It is suggested that up to ⅔ of elderly patients suffer from at least one of these syndromes [8]. In addition, the overlap of these three catabolic states is frequently observed [9]. Sarcopenia is one of the common elements of aging, but its severity is further exacerbated in frailty. Additionally, it is not routinely diagnosed in everyday practice, although it significantly increases the risk of falls and disability in elderly patients. [10].

Sarcopenia and poor nutritional status complement each other in the frail elderly patient phenotype. An improved nutritional status correlates with better performance in elderly patients [11]. Moreover, malnutrition increases the level of multimorbidity [12].

Implementing an interdisciplinary approach and a broad assessment of elderly patients is widely advocated. One of the basic tools for evaluating malnutrition is the Mini Nutritional Assessment (MNA) scale, created in the 1990s. It is based on a questionnaire with a maximum of 30 points. Patients who score less than 24 points are classified as being at risk of malnutrition, and those who score less than 17 points are classified as malnourished. A shortened form of the scale, MNA-SF, is also in use. Despite the passage of time, the MNA scale remains a simple and effective screening tool in the elderly patient population [13]. The SARC-F scale is also a quick tool designed to determine the risk of sarcopenia. It is based on five questions about strength, assistance with walking, rising from a chair, climbing stairs, and falls. The maximum score is 10, and a score above 4 points is considered to be the basis for determining the risk of sarcopenia [14].

In recent years, knowledge and awareness of frailty have improved significantly. Scales for rapid diagnosis of frailty have been enhanced, and risk assessment scales have been created. Moreover, it has been demonstrated that it is possible to prevent frailty and inhibit its development effectively [15]. However, the pathophysiological mechanisms of frailty and its causative factors have not yet been fully elucidated. There is also no consensus on how to manage frail and prefrail patients. A notable knowledge gap concerns the relationship between frailty, malnutrition, and sarcopenia.

Cardiovascular diseases are the leading cause of mortality worldwide. They mainly affect elderly patients. On the other hand, frailty causes the progression of existing cardiovascular diseases. For this reason, studying frailty in cardiology patient cohorts is crucial [15,16]. Those burdened with coronary artery disease (CAD) constitute a special group of patients. Patients with CAD and frailty have a significantly reduced quality of life, and properly implemented treatment improves their functionality. At the same time, patients with CAD are often excluded from studies on new treatment methods [17]; therefore, understanding the overlap between CAD and frailty is a priority.

Considering all these questions, we decided to examine a cohort of elderly CAD patients for frailty. In this study, we aimed to assess differences between patients in different stages of frailty in terms of nutritional status and the concentrations of biomarkers that could potentially indicate malnutrition and sarcopenia. We also aimed to assess the usefulness of scales to evaluate malnutrition and sarcopenia in the frailty population.

## 2. Materials and Methods

### 2.1. Study Design

This study featured an observational cross-sectional design. Therefore, we followed the Strengthening the Reporting of Observational Studies in Epidemiology (STROBE) checklist to report the methods and findings of this study [18]. This is an ancillary analysis of the FRAPICA study (ClinicalTrials.org NCT03209414) [19]. This study was conducted following the Declaration of Helsinki and approved by the Ethics Committee of the Medical University of Silesia (3 October 2017 (KNW/0022/KB1/39/I/17)); (8 February 2022 (PCN/CBN/0022/KB1/39/II/17/22)).

### 2.2. Clinical Setting

The study was conducted in the 2nd Clinical Department of Cardiology in Zabrze, Medical University of Silesia, Poland.

### 2.3. Participants

The study included 170 patients hospitalized between 2022 and 2024 in the 2nd Clinical Department of Cardiology in Zabrze, Poland. The cohort consisted of all consecutively enrolled patients.

#### 2.3.1. Inclusion Criteria

The inclusion criteria for the study included age > 65 years, informed consent to participate in the project, and angiographically confirmed coronary artery disease.

#### 2.3.2. Exclusion Criteria

The exclusion criterion was the lack of consent to participate in this observational study.

### 2.4. Variables and Measurements

During hospitalization, the severity of frailty syndrome was assessed using the Linda Fried scale and the Clinical Frailty Scale.

The degree of frailty was diagnosed using the Frailty Phenotype Score [1] using the following criteria:

- Slowness was assessed by a reduced gait speed over 5 m at usual pace. The patient must repeat the test three times, and the results were averaged. The results were stratified by gender and height.
- Weakness was assessed via the maximal handgrip strength test in the dominant arm. The EH101 electronic hand dynamometer (VETEK AB, Väddö, Sweden) was used. A patient must repeat the test three times, and the maximal value was recorded. The results were stratified by gender and body mass index.
- The Minnesota Leisure Time Activity questionnaire assesses low physical activity. The result is positive when calorie expenditure is lower than 270 kcal/week in women and <383 kcal/week in men. A Microsoft Excel-based template was prepared for rapid questioning and easy calculation of all activities and respective calorie expenditure. Physical activity over the past 12 months was assessed.
- Exhaustion self-reported by a patient was evaluated by answering two questions from the Center for Epidemiologic Studies Depression Scale Revised (CESD-R) scale. The patient must answer the following questions: “How often did you feel like everything you did was an effort in the past week? How often did you feel you could not get going in the past week?” The possible answers are often (≥3 days) or not (when the feeling is present on 0 to 2 days), with the former being a positive answer.
- Weight loss exceeding 10 pounds (approximately 4.5 kg) unintentionally in the past year.

Frailty is recognized if ≥3 out of the 5 criteria are met. Patients in whom 1 or 2 criteria are present are classified as prefrail. Patients who did not meet any of the criteria are marked as robust.

Each patient had a blood sample taken, and the total protein, albumin, and fibrinogen concentrations were determined in the hospital laboratory. Additionally, information on comorbidities, weight, height, and body mass index (BMI) was collected from medical records. Fat-free body mass (FFBM) was assessed using Harpenden’s skinfold caliper and Baty’s body assessment software v. 17 (Baty International Ltd., Burgess Hill, UK). Respiratory parameters, peak expiratory flow (PEF) and forced expiratory volume in one second (FEV1), were measured using an Asmaplan 1 peakflowmeter (Vitalograph, Ennis, Ireland). Additionally, diaphragm thickness was measured with ultrasound [20]. The MNA and SARC-F questionnaire scales were used to assess the overlap of sarcopenia and malnutrition syndromes and their effectiveness in evaluating elderly patients. During hospitalization, each patient was assessed based on the SARC-F questionnaires and the full version of the MNA scale. The following scales were used to evaluate patients: Instrumental Activities of Daily Living (IADL) and Clinical Frailty Scale (CFS).

The MNA and SARC-F questionnaire scales were used to assess the overlap of sarcopenia and malnutrition syndromes and their effectiveness in evaluating elderly patients. The concentrations of albumin, total protein, and fibrinogen were analyzed as biomarkers of the body’s protein metabolism. To analyze the phenomenon of respiratory sarcopenia, the thickness of the diaphragm was measured, as well as the following respiratory parameters: peak expiratory flow (PEF) and forced expiratory second (FEV1).

### 2.5. Statistical Analysis

Having analyzed the data for normality of distribution and equality of variances (Shapiro–Wilk test), we employed both parametric and non-parametric statistics for comparing the robust, prefrail, and frail groups. We used ANOVA for multiple group comparisons of normally distributed quantitative data. Any significant differences identified in the analysis of variance were checked with a Student’s *t*-test corrected with the Bonferroni formula for multiple comparisons. We used Kruskal–Wallis ANOVA to compare non-normally distributed data with multiple comparisons of mean ranks for the samples. The chi-square test with Yates’ correction was used for comparing the frequency data. We have presented the data as means and standard deviations, as medians and interquartile ranges, or as frequency data. Quantitative data presented are unadjusted. We recalculated the values for gender, but the differences were comparable to the unadjusted results. Spearman correlations were calculated for the morphometric, laboratory, and functional parameters of the entire study cohort. As the number of cases in some of the subgroups was small, we used Kruskal–Wallis ANOVA with multiple comparisons of the mean ranks. The robust, prefrail, and frail groups are denoted by the letters R, P, and F in tables. A *p*-value below 0.05 was considered statistically significant. The analysis was performed using Statistica 13.3 software (Tibco, licensed for the Medical University of Silesia).

## 3. Results

### 3.1. Components of the Linda Fried Frailty Definition

The analysis of the average results of the individual components of the Linda Fried scale showed strong correlations between the groups (robust, prefrail, and frail). The strength in the strongest hand expressed in kilograms and the time to cover a distance of 5 m increased with the increasing severity of the frailty. The average value of muscle strength, 23.2 kg, was below the cut-off point for men, who constituted the majority in the frail group. Similarly, the average value of walking speed, 6.13 s, exceeded the norm for both sexes. In addition, patients with frailty syndrome were characterized by the lowest level of physical activity in comparison with the patients from the non-frail and robust groups. In the studied group, a subjective feeling of exhaustion was observed in 38% of patients. In contrast, none of the patients who qualified for the robust group showed signs of exhaustion; this percentage was significantly higher in the prefrail and frail groups (50% and 81%, respectively). Weight loss was noted less frequently: in 19% of patients in the entire group, 21% in the prefrail group, and 62% in the frail group. Similarly, no patient in the robust group noted weight loss.

### 3.2. Demographic and Medical Data

The mean age of the recruited patients was 73.8 years. Of the 170 patients recruited to the study, 53 were classified as robust (31%), 96 as prefrail (57%), and 21 as frail (12%). The groups (robust, prefrail, and frail) were homogeneous in terms of gender. The majority of patients recruited to the study were men (66%). No statistically significant differences in the age of patients were observed between the individual groups (*p* > 0.05). For the separate groups (robust, non-frail, and frail), statistically significant differences were observed in height (*p* < 0.05), but not in weight, fat-free body mass (FFBM), or body mass index (BMI) (*p* > 0.05). Patients did not differ in terms of diaphragm thickness. The most significant percentage of patients were those with hypertension (82%) and hypercholesterolemia (74%). Patients with frailty syndrome showed significantly lower values of respiratory parameters PEF and FEV1, than patients in the robust and prefrail stages (*p* < 0.05). Demographic and medical data of patients are presented in Table 1.

### 3.3. SARC-F, MNA Scales, and Functional Assessment of Patients

Taking into account all patient assessment scales analyzed in the study (Table 2), a statistically significant correlation was noted between the frailty stage and the obtained results (*p* < 0.001 for each scale). The median value of the SARC-F scale for the general, robust, and frail groups was 0. In contrast, patients with frailty syndrome obtained results with a median of 3, which is evidence of a strong association between frailty and sarcopenia. The median scores of the MNA scale in the robust and prefrail groups were similar (27 and 26 points, respectively). In comparison, the median score of the scale in the frail group significantly differed from the other groups (23.5 points, which is the cut-off value for a condition defined as being at risk of malnutrition). Differences in the medians of the CFS and IADL scales showed similar trends. It is worth noting, however, that the median values of the CFS scale in the robust and prefrail groups showed the same values, which questions the validity of using this scale in screening the state defined as prefrail. Multiple comparisons of *p*-value analysis showed no differences between the prefrail and robust stages in any of the analyzed scales.

### 3.4. Concentration of Potential Biomarkers

Among the studied groups, a statistically significant difference was observed in albumin concentrations, with the lowest values in patients diagnosed with frailty (*p* < 0.05). Regarding total protein concentrations, the groups did not differ from each other (NS). Statistical significance was also noted for fibrinogen concentrations, with the highest value for frail patients (Table 3 and Table 4). Multiple-comparison analyses of *p*-values showed no differences in albumin and fibrinogen values between the robust and prefrail groups, i.e., they showed similar values for these parameters. Therefore, the group of patients referred to as frail had the most pronounced disturbances in biomarker concentrations. In contrast, albumin and fibrinogen concentrations were not found to be indicators that differentiate the states referred to as robust and prefrail.

### 3.5. Stratification Analysis According to Frailty Stage, MNA, and SARC-F Score

**Figure 1:**
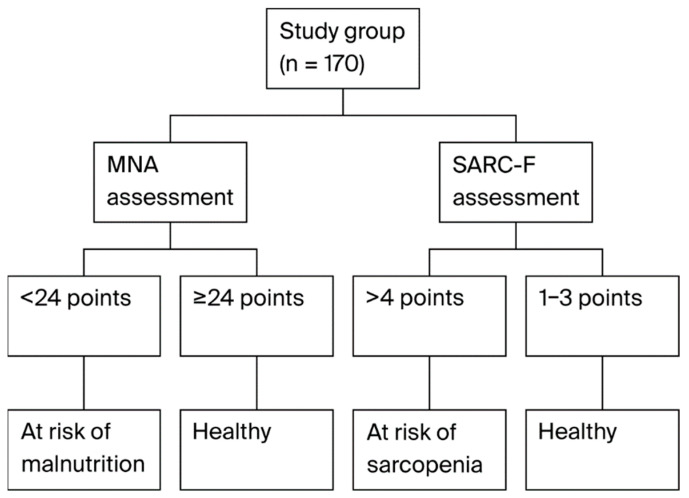
Method of assigning patients to risk groups for sarcopenia and malnutrition.

Analysis of variance allowed for observing differences in the concentration of the studied variables depending on the stage of frailty and the separate groups in the SARC-F and MNA scales, as indicated by the results of the conducted statistical tests (Figure 1). Patients who obtained a score of <3 on the SARC-F scale were classified as not at risk of sarcopenia, and those who received more than 4 points were classified as being at risk of sarcopenia. Patients who obtained a score of more than 24 points on the MNA scale were classified as patients not at risk of malnutrition, and those who received a score below 24 points as being at risk of malnutrition. Out of 21 patients with frailty syndrome, as many as 8 (38%) obtained a score of more than 4 points on the SARC-F scale (at risk of sarcopenia). Moreover, 11 of them (52%) exhibited features indicative of a risk of malnutrition (MNA ≥ 24).

The stratification results for albumin, total protein, and fibrinogen levels are presented in Table 3 and Table 4. It was demonstrated that the SARC-F scale can effectively identify patients with reduced total protein and albumin levels (*p* < 0.01). Similarly, belonging to the group at risk of malnutrition, as expressed in the MNA scale, can help predict a decrease in serum albumin (*p* < 0.01). For fibrinogen, significant differences were observed in the classification according to the Fried frailty scale. Patients with developed frailty were characterized by increased fibrinogen values.

The individual groups that patients belonged to in the MNA and SARC-F scales also influenced the functional results. The SARC-F scale indicates that patients have reduced handgrip strength (*p* < 0.05). The combination of the Fried frailty scale and MNA suggests a group of patients with reduced PEF values, but for FEV1, the only effective predictor is the frailty scale itself. Morphometric and functional assessment of patients stratified by phenotype frailty, SARC-F, and/or MNA classification is presented in Table 5 and Table 6.

Analyzing the entire study group, clear correlations of quantitative values can be observed. Handgrip strength shows strongly positive correlations with respiratory the following parameters: PEF (R = 0.53), FEV1 (R = 0.55), and fat-free body mass (R = 0.60). Fat-free body mass also correlates with PEF (0.40) and FEV1 (0.40). These correlations indicate a possible relationship between the decrease in muscle strength associated with the decline in fat-free body mass, which also affects the strength of respiratory muscles, and thus the functional parameters of the lungs. The remaining correlations are presented in Table 7.

## 4. Discussion

In summary, data on a homogeneous cohort of patients aged 65 and older was collected. Of the 170 patients, 53 were classified as robust (31%), 96 as prefrail (57%), and 21 as frail (12%). Patients classified as frail had significantly reduced serum albumin values and increased fibrinogen values compared to the prefrail and robust groups. Each of the analyzed components of the Fried frailty scale strongly correlates with the severity of frailty. The analysis did not reveal significant differences in the concentration of laboratory parameters and the results of the analyzed scales between the prefrail and robust stages, indicating a need to develop reliable tools for differentiating these states. Stratification of patients according to the risk of malnutrition and sarcopenia, determined using the MNA and SARC-F scales, may help identify patients at the highest risk. At the same time, our results confirm the multidirectional nature of disorders in frailty: declines in physical functions, morphological disorders, respiratory disorders, multimorbidity, and apparent differences in the clinical condition of patients defined as frail.

This study confirms that malnutrition is a significant problem in elderly patients. Albumin levels are strongly correlated with the nutritional status of patients. It also showed a significant decrease in albumin levels in patients with frailty; these results are consistent with previous reports in the literature. Albumin is a potential biomarker of both frailty and sarcopenia, and frail patients have significantly lower albumin levels than their healthy peers, regardless of age [5,6]. In addition, higher albumin levels are correlated with a lower risk of developing frailty [6] and a reduction in total albumin concentration is associated with a decrease in other parameters such as estimated glomerular filtration rate (eGFR) or trace elements [21].

Similarly, patients with higher total protein levels are less likely to develop frailty [6]. The study found no differences in the total protein concentration between the groups. However, belonging to the group at risk of sarcopenia according to the SARC-F scale is associated with a statistically significant decrease in total protein concentration, regardless of the severity of frailty. Nevertheless, albumin, but not total protein, is the most responsive parameter of functional state (strength, speed of movement) and a potential candidate to complete the frailty phenotype.

This study also highlighted a significant difference in fibrinogen concentration between patients with frailty and healthy individuals. Frail patients have increased fibrinogen concentrations, which have been confirmed in the literature. Fibrinogen concentration also increases with age. The underlying cause of this phenomenon may be the pathophysiology of frailty, in which the coagulation state is an integral component and influences catabolic processes (including sarcopenia and malnutrition) [22]. He et al. also found higher fibrinogen values in frail patients, but this was not a biomarker of frailty progression [23]. However, research on the role of fibrinogen in the frailty syndrome is scarce and its effects require further analysis.

An integral element of this study was the analysis of the effectiveness of assessing elderly patients using the MNA and SARC-F scales, which indicated the risk of two main threats to frailty syndrome: sarcopenia and malnutrition. It was shown that patients with frailty syndrome achieve higher values on the SARC-F scale compared to the prefrail and robust groups. On the other hand, they achieve lower results on the MNA scale compared to their peers. Additionally, these scales effectively identify integral disorders resulting from frailty syndrome, including decreased muscle strengths, reduced walking speeds, and decreased respiratory function. Stratification of patients according to their affiliation with high-risk groups, as determined by the MNA, SARC-F, and the Fried frailty scale, may identify groups of patients at a particularly high risk who require intensive preventive and therapeutic measures. These observations have been confirmed in other clinical studies. Patients with reduced walking speeds achieve lower values on the MNA scale [24]. In a study by Li et al., the MNA score was shown to correlate with the skeletal muscle index. This study confirms the results presented in another article, where frailty and malnutrition are associated with sarcopenia [25]. Additionally, it has been suggested that the MNA malnutrition score is a potential predictor of poor physical fitness in elderly patients [11]. On the other hand, others believe that complete assessment using the Global Leadership Initiative on Malnutrition (GLIM), not the abbreviated nutritional assessment (MNA), effectively identifies patients at 5-year risk of sarcopenia [26].

The conclusion regarding the necessity of simultaneous assessment of nutritional status, sarcopenia, and frailty is further supported by the findings of the study by Wei et al., where the simultaneous coexistence of frailty and malnutrition was found to be associated with a worse prognosis and mortality. In contrast, poor nutrition alone without physical frailty is not correlated with an increased risk [27]. On the other hand, the meta-analysis and systematic review by Verlaan et al. indicated that ⅔ of malnourished patients were also frail, and of these patients, only 10% were malnourished [28]. In the present study, more than 50% of patients with frailty syndrome presented features of malnutrition risk. The literature also supports the classification of patients in the study. In the study by Atay et al., similar to this study, most patients were in the prefrail stage, which emphasizes the validity of future actions aimed at fully characterizing this group of patients [29].

In general, recent reports indicate that nutritional status affects the prognosis and condition of patients in every field of medicine. Considering the cohort of the presented study (patients with diagnosed coronary artery disease), the relationship between nutritional status and cardiovascular comorbidities is significant. It has been shown that nutritional status expressed by the MNA scale is an independent predictor of all-cause death and re-hospitalization in patients with heart failure [30]. On the other hand, malnutrition increases the risk of postoperative delirium in elderly patients [31,32], which may be an important finding regarding the invasive and surgical treatment of coronary artery disease and other cardiovascular diseases.

The diagnosis of malnutrition and sarcopenia, therefore, requires intensive effort. Particular attention should be paid to leucine and protein supplementation and resistance training to increase muscle mass and thus improve prognosis [33]. Other analyses indicate the need for supplementation of vitamin D in women and testosterone in men [34]. A personalized diet also improves physical functions and quality of life in malnourished patients [35]. Protein intake above the recommended dietary allowance (RDA) is associated with significant improvement in motor function in elderly patients, which is a protective factor against decreased muscle strength, falls, and disability [36]. On the other hand, the meta-analysis by Coelho-Junior et al. showed that protein intake is not correlated with frailty. Nevertheless, frail patients consume significantly less animal-derived protein than robust patients [37]. Without a doubt, future research should focus on developing standardized protocols and guidelines for preventing and treating sarcopenia and malnutrition in elderly patients.

In the context of scientific research, machine learning can be a valuable resource in developing models for early assessment of malnutrition risk. Such a model was proposed by Liu et al. for a population of terminally ill patients [38]. Another application of emerging technologies may be the use of AI-assisted ultrasonography in the assessment of body composition [39], which may increase the precision of assessing muscle mass loss and indicate patients who are particularly at risk of overlapping sarcopenia and malnutrition. In general, due to the difficulty in decision-making regarding frail patients, AI-based models may prove to be extremely effective in risk assessment and diagnosis of frailty [40]. The solution proposed by Buccheri et al., based on artificial intelligence and decision trees, allows for accurate estimation of muscle mass loss using simple anthropometric data. Such solutions require clinical validation, but they appear to be the future of modern sarcopenia diagnostics [41].

A significant limitation of the presented study is the sample size, which did not allow for drawing more concrete conclusions. Additionally, this study was observational, which impedes clear conclusions, rendering this an underpowered study. Nevertheless, this study provides new information on the relationship between frailty and malnutrition, which should be further explored in future. Additionally, in this study, frailty syndrome was classified using only the Fried frailty scale. The unquestionable advantage of this scale is the simplicity and speed of clinical assessment of the patient. Another advantage is the specification of the state referred to as prefrail, which is potentially reversible [42]. However, more than 50 other tools have been described to diagnose frailty, indicating the need to standardize frailty assessment protocols in clinical trials to compare results and facilitate everyday clinical practice in diagnosing frailty [43].

Another significant limitation of the study was the identification of only three potential biomarkers of frailty. The literature suggests several other immunological, metabolic, hormonal, and genetic molecules. Zeng et al. showed that the fibrinogen-to-albumin ratio may be a potential predictor of frailty [44]. This indicator is poorly studied, indicating further clinical trials are required, especially since it may be a predictor of myocardial infarction in patients undergoing percutaneous coronary interventions [45]. Furthermore, other bodily fluids should be evaluated in the future. Liu et al. demonstrated that the urinary albumin-to-creatinine ratio is significantly correlated with the risk of frailty [46]. This ratio may also indicate the 10-year mortality risk in the elderly population [47]. In general, albumin-based ratios should be further explored in the literature. It has been shown that the albumin–globin ratio, lactate–albumin ratio, blood urea nitrogen–albumin ratio, and fibrinogen–albumin ratio may be important in predicting the risk of heart failure [48], which is common in the elderly population.

## 5. Conclusions

The presented study confirms the uniqueness of patients defined as frail, who exhibit lower muscle strengths, slower gait, reduced physical activity levels, and poorer respiratory parameters. Frailty is also associated with a decreased serum albumin concentration and increased fibrinogen. MNA and SARC-F scales, combined with routine screening of elderly patients with frailty, may allow for effective identification of patients at risk of malnutrition and sarcopenia and with reduced albumin concentrations, which additionally worsens the prognosis. The MNA scale for assessing malnutrition, the SARC-F for evaluating the risk of sarcopenia, and the Fried frailty scale should be included in the management standards for patients hospitalized due to frailty syndrome. In conclusion, we have shown that the development of frailty is multifactorial and that individual components of frailty overlap. Thus, frail patients should be managed by an interdisciplinary team, with particular emphasis on addressing malnutrition and sarcopenia.

## Figures and Tables

**Table 1:** Patients’ demographic and medical data. Normally distributed variables are presented as mean ± standard deviation.

	General *n* = 170	Robust *n* = 53 R	Prefrail *n* = 96 P	Frail *n* = 21 F	Significance
Age (years)	73.8 ± 5.6	72.8 ± 5.2	73.8 ± 5.5	76.3 ± 6.7	NS
Men, *n* (%)	113 (66.5)	42 (79.2)	59 (61.5)	12 (57.1)	*p* < 0.05 (R vs. P)
Weight (kg)	82.6 ± 13.4	82.8 ± 11.0	82.7 ± 14.6	81.8 ± 13.1	NS
Height (cm)	168.6 ± 7.9	170.6 ± 6.5	168.3 ± 8.0	165.3 ± 9.2	*p* < 0.05 (R vs. F)
BMI (kg/cm^2^)	29.1 ± 4.7	28.5 ± 3.7	29.1 ± 4.6	30.5 ± 6.7	NS
FFBM (kg)	56.2 ± 9.2	57.3 ± 8.9	55.8 ± 9.7	55.1 ± 7.2	NS
Diaphragm thickness (mm)	3.7 ± 0.9	3.7 ± 0.8	3.7 ± 0.9	3.5 ± 0.93	NS
PEF (L/min)	316.3 ± 130.1	345.2 ± 135.7	311.9 ± 128.1	266.0 ± 117.0	*p* < 0.05 (R vs. F)
FEV1 (L)	1.95 ± 0.7	2.1 ± 0.7	1.9 ± 0.7	1.5 ± 0.6	*p* < 0.05 (R vs. F, P vs. F)
Hypertension	139 (82%)	46 (87%)	76 (79%)	17 (81%)	NS
Hypercholesterolemia	126 (74%)	41 (77%)	75 (78%)	10 (48%)	*p* < 0.05 (R vs. F, P vs. F)
Diabetes	74 (44%)	21 (40%)	44 (46%)	9 (43%)	NS
Atrial fibrillation	40 (24%)	8 (15%)	24 (25%)	8 (38%)	NS
Renal failure	15 (9%)	4 (8%)	7 (7%)	4 (19%)	NS
COPD/Asthma	25 (15%)	7 (13%)	16 (17%)	2 (1%)	NS
Malignancy	19 (11%)	10 (19%)	7 (7%)	2 (10%)	NS
History of MI	45 (27%)	16 (30%)	24 (25%)	5 (24%)	NS
History of PCI	67 (39%)	23 (43%)	38 (40%)	6 (29%)	NS
History of CABG	18 (11%)	7 (13%)	9 (9%)	2 (10%)	NS

Data presented as mean ± standard deviation or number and percentage; BMI—body mass index; CABG—coronary artery bypass grafting; FFBM—fat-free body mass; MI—myocardial infarction; PCI—percutaneous coronary intervention; PEF—peak expiratory flow; FEV1—forced expiratory volume-one second; NS—non-significant.

**Table 2:** Median scores of MNA, CFS, IADL, and SARC-F scales according to the frailty stage.

	General *n* = 170	Robust *n* = 53 (R)	Prefrail *n* = 96 (P)	Frail *n* = 21 (F)	Significance
SARC-F	0 (0–2)	0 (0–0)	0 (0–0)	3 (2–4)	*p* < 0.001 (R vs. P, R vs. F, P vs. F)
MNA	26.5 (24.5–27.5)	27 (26–28)	26 (24.5–27.5)	23.5 (20–27.2)	*p* < 0.001 (R vs. F, P vs. F)
CFS	3 (2–3)	3 (2–3)	3 (2–3)	4 (3–5)	*p* < 0.001 (R vs. F, P vs. F)
IADL	24 (24–24)	24 (24–24)	24 (24–24)	23 (21–24)	*p* < 0.001 (R vs. F, P vs. F)

MNA—Mini Nutritional Assessment, CFS—Clinical Frailty Scale, IADL—Instrumental Activities of Daily Living. Data presented as median and interquartile ranges. P, R, and F denote robust, prefrail, and frail patients, respectively.

**Table 3:** Blood plasma protein, albumin, and fibrinogen concentrations stratified by phenotype frailty and SARC-F classification.

		Robust	Prefrail	Frail	Significance
Total protein, g/L (X ± SEM)	SARC-F 0–3	66.7 ± 0.7 (*n* = 49)	66.7 ± 0.5 (n = 86)	65.9 ± 1.4 (*n* = 13)	NS (frailty) *p* < 0.01 (SARC-F)
	SARC-F 4–10	64.3 ± 2.2 (*n* = 5)	62.5 ± 1.6 (n = 10)	62.6 ± 1.7 (*n* = 8)	
Albumin, g/L (X ± SEM)	SARC-F 0–3	42.9 ± 0.5 (*n* = 49)	42.1 ± 0.4 (n = 85)	40.6 ± 1.0 (*n* = 13)	NS (frailty) *p* < 0.01 (SARC-F)
	SARC-F 4–10	39.5 ± 1.7 (*n* = 5)	39.5 ± 1.2 (n = 10)	37.6 ± (*n* = 8)	
Fibrinogen, g/L (X ± SEM)	SARC-F 0–3	3.1 ± 0.1 (*n* = 49)	3.1 ± 0.1 (n = 85)	3.4 ± 0.2 (*n* = 13)	*p* < 0.05 (frailty) NS (SARC-F)
	SARC-F 4–10	3.3 ± 0.3 (*n* = 5)	3.1 ± 0.2 (n = 10)	3.8 ± 0.2 (*n* = 8)	

Kruskal–Wallis ANOVA with one-way classification was used for analysis; X ± SEM—mean and standard error of the mean, NS—non-significant.

**Table 4:** Blood plasma protein, albumin, and fibrinogen concentrations stratified by phenotype frailty and MNA classification.

		Robust	Prefrail	Frail	Significance
Total protein, g/L (X ± SEM)	MNA ≥ 24	66.5 ± 0.7 (*n* = 48)	66.6 ± 0.6 (n = 75)	65.7 ± 1.6 (*n* = 10)	NS (frailty) NS (MNA)
	MNA < 24	66.3 ± 2.1 (*n* = 6)	64.9 ± 1.1 (n = 20)	63.7 ± 1.5 (*n* = 11)	
Albumin, g/L (X ± SEM)	MNA ≥ 24	42.6 ± 0.5 (*n* = 48)	42.5 ± 0.4 (n = 74)	42.2 ± 1.2 (*n* = 10)	*p* < 0.05 (frailty) *p* < 0.01 (MNA)
	MNA < 24	42.5 ± 1.5 (*n* = 6)	39.5 ± 0.8 (n = 20)	37.8 ± 1.1 (*n* = 11)	
Fibrinogen, g/L (X ± SEM)	MNA ≥ 24	3.1 ± 0.1 (*n* = 48)	3.1 ± 0.1 (n = 74)	3.7 ± 0.2 (*n* = 10)	*p* < 0.05 (frailty) NS (MNA)
	MNA < 24	3.2 ± 0.3 (*n* = 6)	3.1 ± 0.2 (n = 20)	3.5 ± 0.2 (*n* = 11)	

Kruskal–Wallis ANOVA with one-way classification was used for analysis; X ± SEM—mean and standard error of the mean, NS—non-significant.

**Table 5:** Morphometric and functional assessment of patients stratified by phenotype frailty and SARC-F classification.

		Robust	Prefrail	Frail	Significance
Body weight, kg (X ± SEM)	SARC-F 0–3	85.0 ± 2.1 (*n* = 49)	82.2 ± 0.5 (*n* = 86)	81.6 ± 4.2 (*n* = 13)	NS (frailty) NS (SARC-F)
	SARC-F 4–10	77.0 ± 6.7 (*n* = 5)	86.8 ± 4.7 (*n* = 10)	82.0 ± 5.3 (*n* = 8)	
Fat-free body mass, kg (X ± SEM)	SARC 0–3	57.9 ± 1.3 (*n* = 49)	55.3 ± 1.0 (*n* = 85)	55.1 ± 2.5 (*n* = 13)	NS (frailty) NS (SARC-F)
	SARC 4–10	50.8 ± 4.1 (*n* = 5)	60.0 ± 2.9 (*n* = 10)	55.1 ± 3.2 (*n* = 8)	
Diaphragm thickness, mm (X ± SEM)	SARC 0–3	3.7 ± 0.1 (*n* = 49)	3.8 ± 0.1 (*n* = 85)	3.6 ± 0.2 (*n* = 13)	NS (frailty) NS (SARC-F)
	SARC 4–10	4.1 ± 0.4 (*n* = 5)	3.0 ± 0.3 (*n* = 10)	3.2 ± 0.3 (*n* = 8)	
Gait speed, s/5 m (X ± SEM)	SARC-F 0–3	3.7 ± 0.6 (*n* = 49)	4.4 ± 0.5 (*n* = 49)	10.9 ± 1.2 (*n* = 49)	*p* < 0.01 (frailty) NS (SARC-F)
	SARC-F 4–10	5.2 ± 1.9 (*n* = 5)	7.1 ± 1.4 (*n* = 5)	7.6 ± 1.5 (*n* = 5)	
Handgrip strength, kg (X ± SEM)	SARC-F 0–3	36.3 ± 1.3 (*n* = 49)	29.1 ± 1.0 (*n* = 86)	23.7 ± 2.5 (*n* = 13)	*p* < 0.05 (frailty) *p* < 0.05 (SARC-F)
	SARC-F 4–10	26.2 ± 4.0 (*n* = 5)	27.2 ± 2.9 (*n* = 10)	22.3 ± 3.2 (*n* = 8)	
PEF, L/min (X ± SEM)	SARC-F 0–3	355.0 ± 18.9 (*n* = 46)	311.1 ± 14.2 (*n* = 82)	301.0 ± 35.6 (*n* = 13)	NS (frailty) NS (SARC-F)
	SARC-F 4–10	261.0 ± 57.4 (*n* = 5)	331.5 ± 45.4 (*n* = 8)	209.0 ± 45.4 (*n* = 8)	
FEV1, L/sec (X ± SEM)	SARC-F 0–3	2.1 ± 0.1 (*n* = 46)	1.9 ± 0.1 (*n* = 82)	1.7 ± 0.2 (*n* = 13)	*p* < 0.01 (frailty) NS (SARC-F)
	SARC-F 4–10	1.9 ± 0.3 (*n* = 5)	2.3 ± 0.2 (*n* = 8)	1.3 ± 0.3 (*n* = 8)	

PEF—peak expiratory flow; FEV1—forced expiratory flow in one second; X ± SEM—mean and standard error of the mean. Kruskal–Wallis ANOVA with one-way classification was used for analysis, NS—non-significant.

**Table 6:** Morphometric and functional assessment of patients stratified by phenotype frailty and MNA classification.

		Robust	Prefrail	Frail	Significance
Body weight, kg (X ± SEM)	MNA ≥ 24	82.7 ± 2.1 (*n* = 48)	83.4 ± 1.7 (*n* = 75)	85.8 ± 4.7 (*n* = 10)	NS (frailty) NS (MNA)
	MNA < 24	96.7 ± 6.1 (*n* = 6)	79.7 ± 3.3 (*n* = 20)	78.1 ± 4.5 (*n* = 11)	
Fat-free body mass, kg (X ± SEM)	MNA ≥ 24	57.4 ± 1.3 (*n* = 48)	56.1 ± 1.1 (*n* = 75)	56.9 ± 2.9 (*n* = 10)	NS (frailty) NS (MNA)
	MNA < 24	55.9 ± 3.8 (*n* = 6)	54.6 ± 2.1 (*n* = 20)	53.5 ± 2.8 (*n* = 11)	
Diaphragm thickness, mm (X ± SEM)	MNA ≥ 24	3.7 ± 0.1 (*n* = 48)	3.7 ± 0.1 (*n* = 73)	3.2 ± 0.3 (*n* = 10)	NS (frailty) NS (MNA)
	MNA < 24	3.8 ± 0.3 (*n* = 6)	3.6 ± 0.2 (*n* = 20)	3.7 ± 0.3 (*n* = 11)	
Gait speed, s/5 m (X ± SEM)	MNA ≥ 24	3.8 ± 0.6 (*n* = 48)	4.3 ± 0.5 (*n* = 75)	13.1 ± 1.3 (*n* = 10)	*p* < 0.001 (frailty) NS (MNA)
	MNA < 24	4.0 ± 1.7 (*n* = 6)	6.0 ± 0.9 (*n* = 20)	6.5 ± 1.3 (*n* = 11)	
Handgrip strength, kg (X ± SEM)	MNA ≥ 24	35.8 ± 1.3 (*n* = 48)	29.3 ± 1.1 (*n* = 75)	22.2 ± 2.9 (*n* = 10)	*p* < 0.001 (frailty) NS (MNA)
	MNA < 24	32.0 ± 3.7 (*n* = 6)	27.9 ± 2.1 (*n* = 20)	24.0 ± 2.8 (*n* = 11)	
PEF, L/min (X ± SEM)	MNA ≥ 24	348.6 ± 19.0 (*n* = 45)	326.3 ± 15.1 (*n* = 71)	206.6 ± 40.2 (*n* = 10)	NS (frailty) NS (MNA)
	MNA < 24	324.8 ± 52.0 (*n* = 6)	270.0 ± 30.0 (*n* = 18)	319.9 ± 38.4 (*n* = 11)	
FEV1, L/s (X ± SEM)	MNA ≥ 24	2.1 ± 0.1 (*n* = 45)	2.0 ± 0.1 (*n* = 71)	1.3 ± 0.2 (*n* = 10)	*p* < 0.05 (frailty) NS (MNA)
	MNA < 24	1.6 ± 0.3 (*n* = 6)	1.9 ± 0.2 (*n* = 18)	1.8 ± 0.2 (*n* = 11)	

PEF—peak expiratory flow; FEV1—forced expiratory flow in one second; X ± SEM—mean and standard error of the mean. Kruskal–Wallis ANOVA with one-way classification was used for analysis, NS—non-significant.

**Table 7:** Correlation matrix of morphometric, laboratory, and functional parameters for the entire study population.

	Weight (kg)	Total Protein (g/L)	Albumin (g/L)	Fibrinogen (g/L)	Fat-Free Body Mass (kg)	Gait Speed (s/5 m)	Handgrip Strength (kg)	PEF (L/min)	FEV1 (L)
Weight (kg)	1.000								
Total Protein (g/L)	0.191 *	1.000							
Albumin (g/L)	0.247 *	0.660 *	1.000						
Fibrinogen (g/L)	−0.023	−0.060	−0.190 *	1.000					
Fat-Free Body Mass (kg)	0.664 *	0.118	0.093	−0.077	1.000				
Gait Speed (s/5 m)	0.049	0.070	0.012	0.182 *	0.013	1.000			
Handgrip Strength (kg)	0.269 *	0.167 *	0.190 *	−0.147	0.604 *	−0.134	1.000		
PEF (L/min)	0.214 *	0.070	0.051	−0.184 *	0.402 *	−0.128	0.528 *	1.000	
FEV1 (L)	0.149	0.065	0.106	−0.229 *	0.404 *	−0.111	0.549 *	0.749 *	1.000
Diaphragm Thickness (mm)	0.115	0.237 *	0.207 *	−0.075	0.050	−0.128	0.152	0.159 *	0.132

* The correlation is significant.

## Data Availability

The original contributions presented in the study are included in the article; further inquiries can be directed to the corresponding authors.

## Abbreviations

The following abbreviations are used in this manuscript:

MNA	Mini Nutritional Assessment
MNA-SF	Mini Nutritional Assessment Short Form
FRAPICA	Frailty Syndrome in Daily Practice of Interventional Cardiology Ward
BMI	Body Mass Index
FFBM	Fat-Free Body Mass
PEF	Peak Expiratory Flow
FEV1	Forced Expiratory Volume-One Second
IADLs	Instrumental Activities of Daily Living
CFS	Clinical Frailty Scale
COPD	Chronic Obstructive Pulmonary Disease
MI	Myocardial Infarction
PCI	Percutaneous Coronary Intervention
CABG	Coronary Artery Bypass Grafting
